# Case report: Simultaneous measurement of intracranial pressure and lumbar intrathecal pressure during epidural patch therapy for treating spontaneous intracranial hypotension syndrome. Spontaneous intracranial hypotension or spontaneous intraspinal hypovolume?

**DOI:** 10.3389/fneur.2024.1308462

**Published:** 2024-03-21

**Authors:** Nicolas Engrand, Quentin Salardaine, Jean-Philippe Desilles, Cécile Echard, Pierre Bourdillon, Marc Williams, Guillaume Baille

**Affiliations:** ^1^Neuro-Intensive Care Unit-Anesthesiology, Rothschild Foundation Hospital, Paris, France; ^2^Neurology Department, Delafontaine Hospital, Saint-Denis, France; ^3^Interventional Neuroradiology Department, Rothschild Foundation Hospital, Paris, France; ^4^Neurosurgery Department, Rothschild Foundation Hospital, Paris, France; ^5^Diagnostic Neuroradiology Department, Rothschild Foundation Hospital, Paris, France

**Keywords:** subacute subdural hematoma, spontaneous intracranial hypotension, epidural patch, bithalamic ischemia, case report

## Abstract

**Objectives:**

Spontaneous intracranial hypotension (SIH) is frequently complicated by subacute subdural hematoma (SDH) and more rarely by bilateral thalamic ischemia. Here, we report a case of SIH-related SDH treated with three epidural patches (EPs), with follow-up of the intracranial pressure and lumbar intrathecal pressure.

**Methods:**

A 46-year-old man presented bilateral thalamic ischemia, then a growing SDH. After failure of urgent surgical evacuation, he underwent three saline EPs, two dynamic myelography examinations and one digital subtraction angiography–phlebography examination. However, because of no dural tear and no obstacle to the venous drainage of the vein of Galen, no therapeutic procedure was available, and the patient died.

**Results:**

The case exhibited a progressive increase in the transmission of lumbar intrathecal pressure to intracranial pressure during the three EPs. The EPs may have successfully treated the SIH, but the patient did not recover consciousness because of irreversible damage to both thalami.

**Conclusion:**

Clinicians should be aware of the bilateral thalamic ischemia picture that may be the presenting sign of SIH. Moreover, the key problem in the pathophysiology of SIH seems to be intraspinal and intracranial volumes rather than pressures. Therefore, intracranial hypotension syndrome might actually be an intraspinal hypovolume syndrome.

## Introduction

Spontaneous intracranial hypotension (SIH) is frequently complicated by subacute subdural hematoma (SDH) (1–4) and more rarely bilateral thalamic ischemia (5–8). The management of this condition is poorly codified and is often based on surgical evacuation of the SDH. However, this therapeutic approach remains controversial because of the paradoxical nature of removing the intracranial volume in the context of pre-existing hypotension. Consequently, a growing number of authors now recommend treating SIH-related SDH with a blood patch, targeted to a possible dural tear or not (1, 4, 8–12). The management of rare bilateral thalamic strokes is even less known.

Here, we present a case of SIH complicated by SDH, with follow-up of the intracranial pressure (ICP) and lumbar intrathecal pressure (LITP) during the etiological therapeutic management (epidural patches [EPs]). The case allows for further understanding treatment.

## Case report

**Figure 1:**
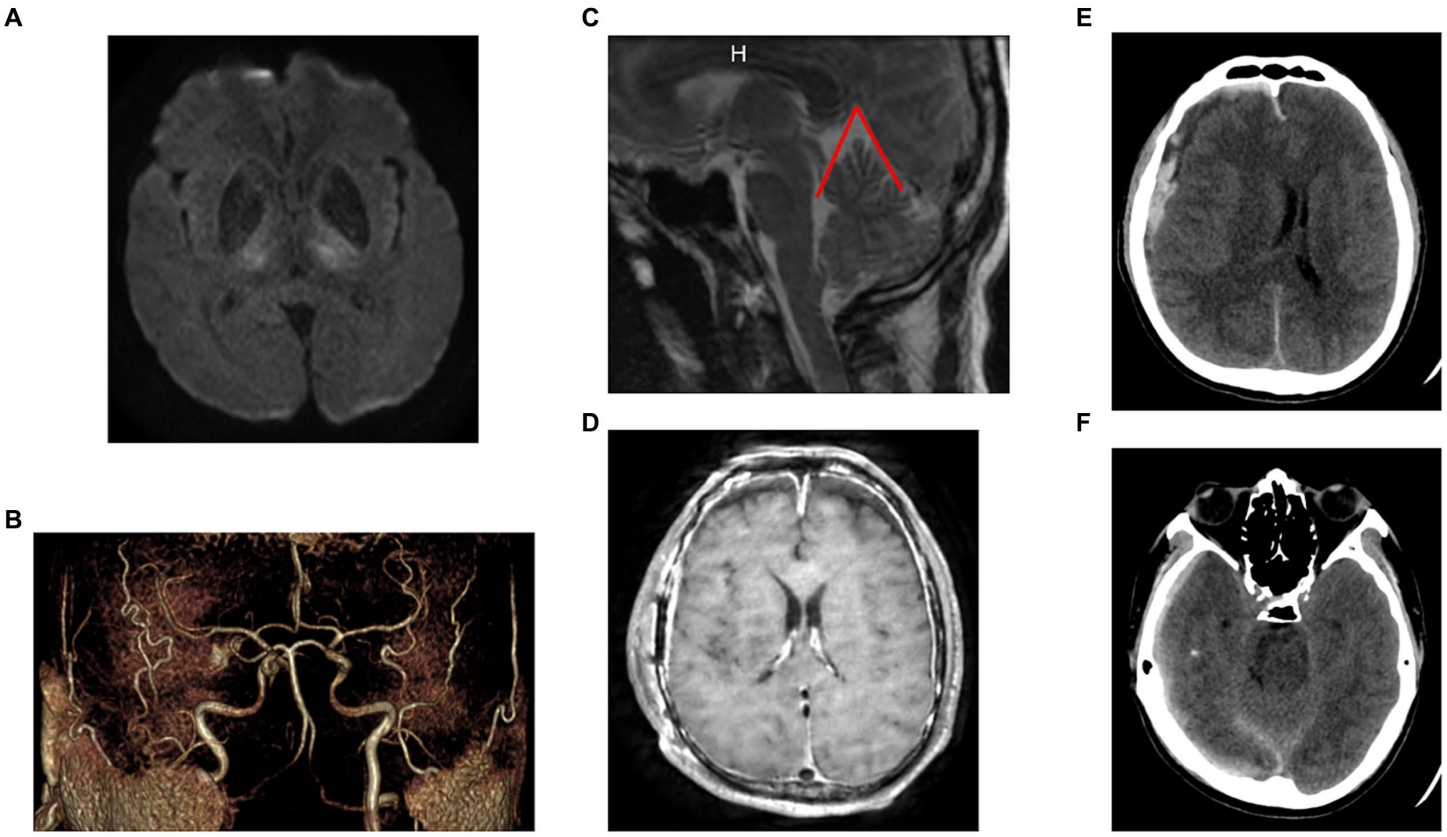
Initial imaging. **(A)** Initial [day 4 (D4)] MRI (axial diffusion b2000 sequence): bilateral thalamic ischemia. **(B)** Initial (D4) MRI (3D arterial time-of-flight image): no arterial amputation favoring ischemic stroke. **(C)** Initial (D4) MRI (sagittal view of a T2-weighted sequence): evidence of posterior sagging of the brain and brainstem, with closure of the angle between the protuberance and the diencephalon, verticalization of the cerebellar tent, herniation of the cerebellar amygdala, favoring the SIH diagnosis. The angle between the vein of Galen and the right sinus was measured at 52° (angle marked in red). **(D)** D17 MRI (axial T1-weighted sequence): significant and diffuse pachymeningeal enhancement after contrast agent injection and markedly distended dural venous sinuses, favoring the SIH diagnosis. **(E)** D0 CT-scan (no contrast agent). Evidence of a 13-mm-thick heterogenous subdural collection in the right convexity with significant midline deviation. **(F)** D0 CT-scan: No uncal herniation, favoring no intracranial hypertension (SDH, subacute subdural hematoma; SIH, spontaneous intracranial hypotension; D0: day of neurosurgical evacuation of the SDH).

A 40-50 year-old patient with no known medical condition other than chronic alcoholism and no usual treatment was referred to the stroke unit for severe aphasia. The patient was slightly isolated socially, and next-of-kin interrogations revealed orthostatic headache and walking disturbance for 4 months. The initial examination revealed delirium, aphasia, and spastic hypertonia of all four limbs. Pulse oxygen saturation and blood glucose level were normal. Immediate brain MRI highlighted bilateral thalamic diffusion and FLAIR hypersignals, with apparent diffusion coefficient hyposignals, associated with thin, chronic, right convexity SDH (Figure 1A). Arterial 3D time-of-flight sequences did not show arterial amputations consistent with ischemic stroke (Figure 1B), and no cerebral venous (sinus) thrombosis was evidenced.

**Figure 2:**
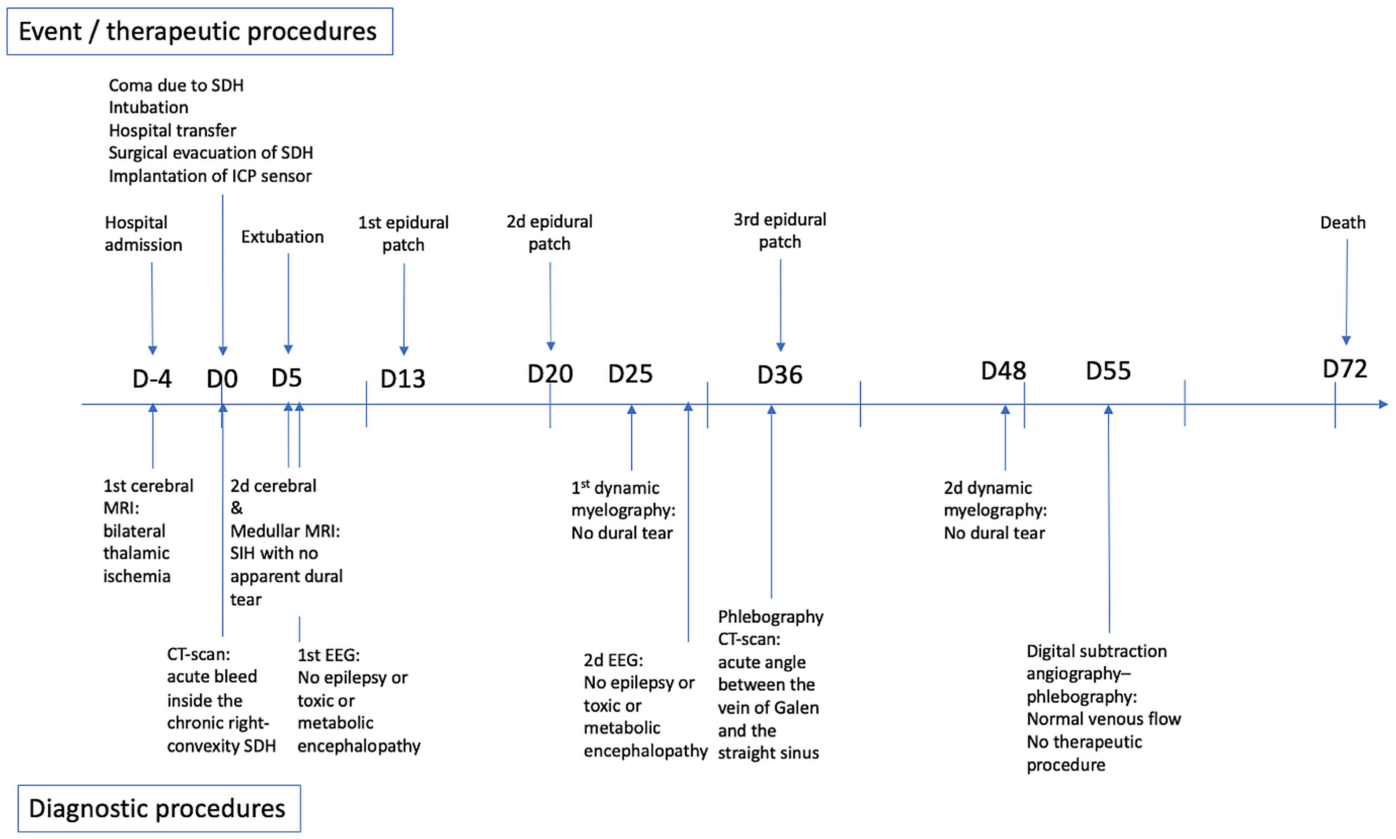
Timeline of diagnostic and therapeutic procedures.

**Figure 3:**
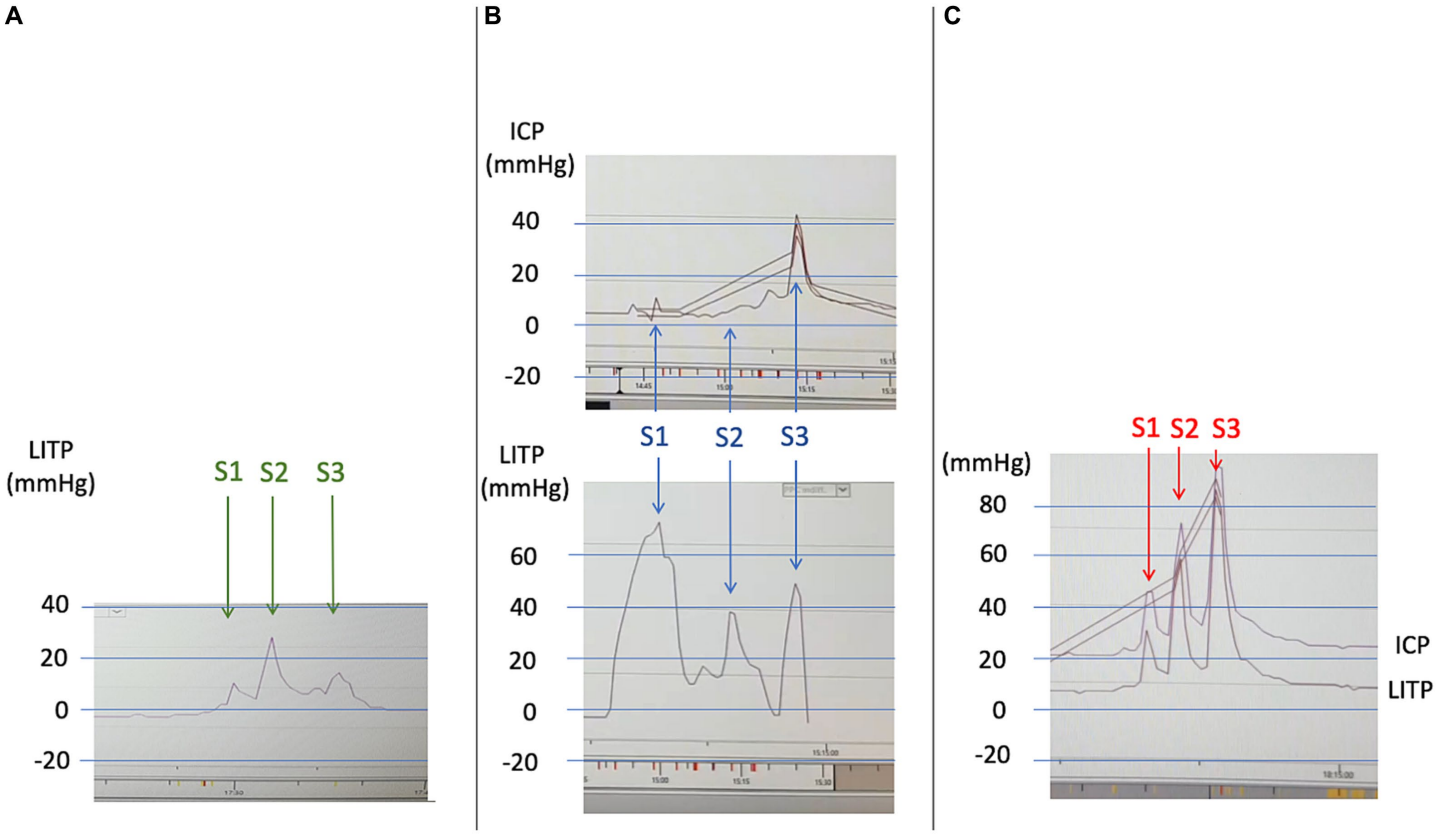
LITP and ICP curves during the three saline epidural patches (identical scales). Each procedure included an initial measurement of LITP at L3-L4. The epidural patch treatment involved three 20-ml syringe injections into the epidural space at L2-L3 (marked by arrows and annotated S1, S2, S3). **(A)** First epidural patch (D13). Three epidural injections of 20-mL volume led to a moderate increase in LITP (max 25 mmHg), with no increase in ICP (curve not recorded). **(B)** Second epidural patch (D20). Three epidural injections of 20-mL volume led to a transient but significant increase in LITP (max 75 mmHg), with an increase in ICP only during the third injection (max 45 mmHg). **(C)** Third epidural patch (D36). Three epidural injections of 20-mL volume resulted in a major and strictly parallel increase in LITP and ICP during all three injections (max 85 mmHg). Thus, the three epidural patches led to a progressive increase in LITP and ICP as well as transmission from LITP to ICP. This phenomenon probably indicates that the intraspinal and intracranial compliances became lower, even if the pressures themselves remained low (apart from their transient increase during the injections). Hence, volume deficit of the intraspinal compartment was probably filled by the first epidural injections and the dural tear was treated (LITP, lumbar intrathecal pressure; ICP, intracranial pressure).

Biological work-up revealed HIV infection associated with lymphopenia, with no associated infections. SARS-CoV-2 PCR results for a nasal swab were negative. Four days after hospital admission, the patient became comatose (Glasgow coma score 6). An urgent brain CT scan revealed an acute bleed inside the chronic right-convexity SDH (13 mm total thickness) causing a subfalcine herniation without associated uncal herniation (Figures 1E,F). After intubation, the patient underwent surgery for evacuation of the SDH. During the surgery, the brain did not re-expand to the dura, and venous bleeding was observed (bridging veins). An intracranial pressure sensor was implanted for monitoring (Pressio®2, Sophysa, Orsay France). During the next days, despite the cessation of sedation on day one (D1) and extubation on D5 postoperatively, the patient did not recover contact with the environment (Glasgow coma score 10), exhibiting fluctuating eye opening and severe spastic tetraparesis. A review of the MRI (Figures 1C,D) and the ICP measurements confirmed the diagnosis of severe SIH. A spinal MRI revealed cervico-arthrosis causing posterior spinal cord compression at C6-C7 with a mild centromedullary T2-weighted hypersignal but no apparent dural tear. Electroencephalography did not reveal epileptic activity or toxic or metabolic encephalopathy. Biological investigations ruled out the differential diagnoses: viral and bacterial meningo-encephalitis, auto-immune encephalitis, hyperammonemia, toxic encephalitis, and vitamin B1 deficiency. Although immunophenotyping revealed a CD4 lymphocyte count of 4/mm3 with a viral load of 5 log, the diagnosis of HIV-specific encephalitis was not retained because of the unfavorable radiological picture. No opportunistic infections were found. A summary of the biological investigations is in Supplementary Table 1. Figure 2 shows a timeline of diagnostic and therapeutic procedures. Intensive care unit follow-up was uneventful, with satisfactory airway control by the patient, although two nosocomial infections occurred (ventilator-associated pneumonia and bacteremia), which were resolved with antibiotics. SIH treatment initially consisted of the Trendelenburg position, with a moderate effect on ICP (values from 0 to 5 mmHg vs. −3 to 0 mmHg in the semi-seated position), but with no substantial effect on consciousness. Then, three saline EPs were performed, on D13, D20 and D36. Each procedure included an initial measurement of LITP at L3-L4 with a lumbar puncture needle and tubing fitted with a pressure transducer, to verify that it was low and to perform the various intrathecal tests for etiologic purposes. The EPs consisted of three 20-ml syringe injections into the epidural space at L2-L3 while measuring LITP and ICP (Figure 3). Saline injection was preferred to blood injection because of the high HIV viremia. The first lumbar puncture was very difficult, with the need to suck with the syringe to obtain less than 1 mL of cerebrospinal fluid (CSF) (hence, no laboratory analysis could be performed), whereas the following punctures were considered “easy” (allowing for laboratory analysis).

Because of the persistence of the patient’s lack of communication and low ICP (continuously ≤5 mmHg), two dynamic myelography examinations were performed on D25 and D48 to search for a dural tear that could have been surgically repaired (Supplementary Figures 1A,B). However, no extra-thecal CSF leak was found. During the first dynamic myelography, ICP immediately and transiently increased from 2 to 80 mmHg, and the optic nerve sheath diameters measured on ultrasonography immediately dilated from 6.4 to 7.7 mm (Supplementary Figures 1C,D). Finally, in view of the bilateral thalamic ischemia and the sharp angle between the vein of Galen and the straight sinus on a D35 phlebography–CT scan (measured at 12°, Supplementary Figures 1E,F), digital subtraction angiography–phlebography was performed on D55 to search for an obstacle to the venous drainage. However, venous flow was normal, and no therapeutic procedure could be performed.

Unfortunately, 2 months after the aphasia onset, the patient remained in a minimally conscious state and died after active therapy was discontinued on D72.

## Discussion

This case report is the first to describe simultaneous ICP and LITP measurements during EP therapy for SIH. SIH is defined by CSF opening pressure ≤ 60 mm H_2_O (3). In most cases, SIH leads to mild symptoms (typically orthostatic headaches), with good outcome. However, spontaneous SDH is a common complication of SIH (up to 20–45%), due to rupture of bridging veins between the dura and surface of the brain, and can be frequently recurrent (1–3, 9). In the present case, the diagnosis of bilateral thalamic arterial infarct was first considered, but lesions encountered in such cases involve more the anterior and lateral parts of the thalamus supplied by the paramedian thalamic artery (13). Bilateral thalamic lesions are a rare radiological feature of SIH and could be linked to a closing angle between the vein of Galen and the straight sinus compromising the venous drainage of thalami and resulting in vasogenic edema (5–8). In addition, a narrower angle between the vein of Galen and the straight sinus was found associated with poor response to an epidural blood-patch (50 ± 16° vs. 67 ± 26°) (14). This angle could even close if the blood patch were ineffective.

In our case, the first or second EP may have treated the SIH, which would explain why subsequent etiologic investigations did not reveal a dural tear or venous obstruction. However, the patient would not have recovered consciousness because of the irreversible damage to both thalami caused by the venous ischemia. The progressive increase in transmission of LITP to ICP observed during the three EPs argues for this hypothesis. The absence of pressure transmission from the lumbar to the cranial compartment during the first epidural injections (Figures 3A,B) revealed an abnormally high compliance of the spinal compartment. Then, the superposition of pressure peaks between the two compartments during the last injections (Figure 3C) revealed that compliances had normalized and were balanced between the two compartments. Variations in intraspinal and intracranial volumes rather than pressures probably determined the respective compliances and symptoms of SIH, as previously reported (15). The hypothesis of this sequence is all the more likely because the first lumbar puncture was difficult due to lack of pressure in the intrathecal space, whereas dynamic myelography, after the leak had probably been sealed, resulted in immediate dilation of the optic nerve sheath diameters.

A recent article reported two cases of ICP measurement during successful blood EP treatment of SIH complicated by SDH (16). Similarly, epidural injections led to an increase in ICP, but this was treated in turn by opening the previously placed intracranial subdural drains. Another study also showed that treating SIH with blood EP led to an increase in optic nerve sheath diameters (which indirectly reflects ICP) and even suggested that this increase was a marker of treatment efficacy (17).

**Figure 4:**
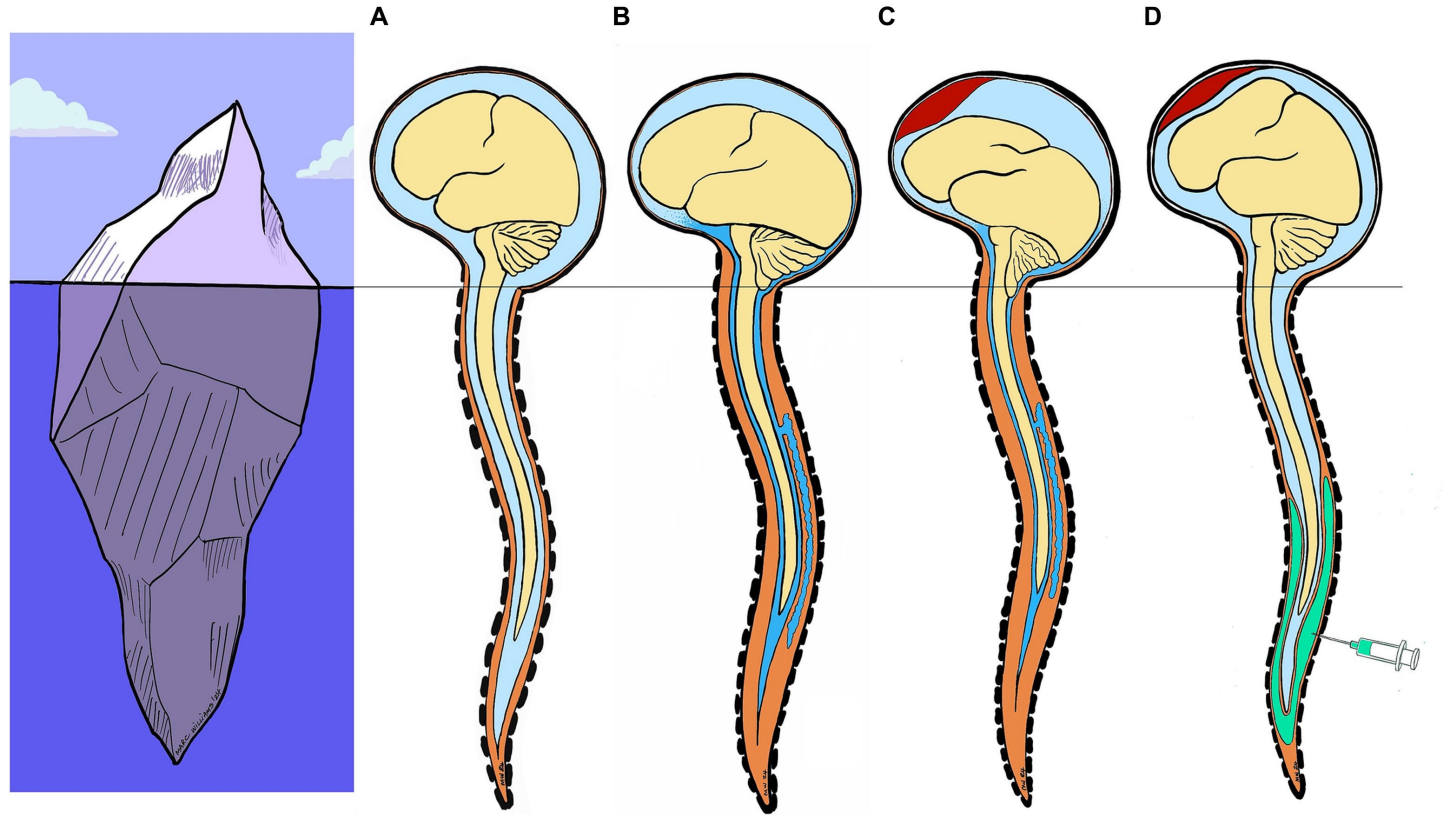
Model of SIH pathophysiology and epidural patch action. Iceberg analogy: the melting of the glacier (brain) on the surface (base of skull) follows a previous underwater event (spinal cord) **(A)** Normally, the brain is suspended in its CSF pool **(B)** In the event of SIH, the CSF pool empties and the brain tilts backward onto the skull base. **(C)** Subdural hematoma is a common complication of SIH, due to rupture of bridging veins between dura and surface of the brain. **(D)** SIH treatment with an EP seems to act by 1) interrupting the process of spinal transdural siphoning (extradural pressure becoming greater than subarachnoid pressure; the pressure effect), and 2) refilling the fluid volume deficit in the entire intraspinal compartment (the volume effect). Injecting a volume of fluid into the spinal epidural space flushes CSF from the perimedullary subarachnoid compartment into the continuous periencephalic subarachnoid compartment, thus restoring the brain’s floating pool and enabling the brain to tilt forward again. (CSF, cerebrospinal fluid; SIH, spontaneous intracranial hypotension; EP, epidural patch).

To synthesize our pathophysiological hypothesis, one must first remember that normally, the brain is suspended in its CSF pool. In the event of SIH, the pool empties and the brain tilts backward onto the skull base (Figure 4). SIH treatment with an EP seems to act by (1) interrupting the process of spinal transdural siphoning (extradural pressure becoming greater than subarachnoid pressure; the pressure effect), rather than by anatomically sealing a breach (18), and (2) refilling the fluid volume deficit in the entire intrarachid compartment (also known as the “spinal” compartment, which includes the intradural and the epidural space; the volume effect). Thus, injecting a volume of fluid into the spinal epidural space flushes CSF from the perimedullary subarachnoid compartment into the continuous periencephalic subarachnoid compartment, thereby restoring the brain’s floating pool and enabling the brain to tilt forward again (Figure 4). This is followed by a significant transient increase in ICP lasting from a few seconds to a few minutes, shown by the ICP curves (Figure 3), as previously reported (16, 18). In fact, this is what anesthetists unknowingly do when treating a post-peridural dural breach with a blood patch, stopping the injection just as the headache appears. Thus, headache probably indicates that CSF has shifted from the spinal compartment into the cranial compartment, hence leading to transient intracranial hypertension. This hypothesis would also explain why the EP can be effective regardless of the spinal injection level (4, 10–12) and the nature of the fluid, blood or saline (18, 19).

We suggest an algorithm for the investigation and therapeutic management of SDH with SIH (Supplementary Figure 2): the therapeutic and etiological strategy would be progressively more invasive, starting with an initial assessment using MRI sequences of the skull and spine, followed by non-targeted lumbar EPs, which may be repeated once or twice, and finally, in the event of failure, dynamic myelography in search of a persistent extensive tear, with surgical repair if necessary, but with no indication for surgical removal of subacute SDH except in the event of coma with uncus herniation.

Our case report has three main limitations. First, a peri-medullary breach was not definitively established, but the diagnosis of SIH was not in doubt (clinical history, MRI findings, ICP and LITP always low). Second, the narrowing of the cervical canal at C6-C7 could have affected pressure transmission between the lumbar sac and cranium. However, the narrowing of the cervical canal was limited to its posterior part, and the two dynamic myelograms showed no slowing of contrast agent diffusion toward the rostral level. In any case, the transmission between the two pressure measurements could have been hindered, with an underestimation rather than an overestimation of the effect we have documented. Third, other associated conditions could explain the bithalamic lesions and the lack of conscious recovery. Although acute Wernicke’s encephalopathy, osmotic demyelination or cerebral venous sinus thrombosis do not seem to be involved given the overall clinical, biological and radiological picture, cerebral lymphoma or encephalitis (HIV, West Nile virus, Japanese encephalitis or tick-borne encephalitis) were not formally ruled out. Nevertheless, the diagnosis of SIH and the evolution of ICP and LITP curves remain relevant, illustrating the pathophysiology of this syndrome.

In conclusion, clinicians should be aware of the bilateral thalamic ischemia picture that may be the presenting sign of a SIH. Also, the spinal compartment has an established central role in the pathophysiology of SIH, but the key problem may be intraspinal and intracranial volumes rather than pressures. Therefore, intracranial hypotension syndrome could actually be intraspinal hypovolume syndrome.

## Data availability statement

The original contributions presented in the study are included in the article/Supplementary material, further inquiries can be directed to the corresponding author.

## Ethics statement

Ethical approval was not required for the studies. The studies were conducted in accordance with the local legislation and institutional requirements. Written informed consent for participation was not required from the participants or the participants’ legal guardians/next of kin in accordance with the national legislation and institutional requirements because the patient has died. The deceased patient’s next of kin did not object to publication of the case study after the information had been provided.

## Author contributions

NE: Investigation, Writing – original draft, Conceptualization, Data curation, Formal analysis, Validation. QS: Data curation, Investigation, Writing – original draft, Writing – review & editing. J-PD: Investigation, Writing – review & editing. CE: Writing – review & editing, Writing – original draft. PB: Writing – review & editing. GB: Writing – review & editing, Investigation, Writing – original draft. MW: Writing – original draft, Writing – review & editing.

## Supplementary material

Supplementary Figure 1 Subsequent imaging. **(A)** First dynamic myelography (D25). No extra-thecal CSF leak found. **(B)** CT-scan myelography (D25). The patient was transferred for CT immediately after the dynamic myelography was performed, without further contrast injection into the intrathecal space. No extra-thecal CSF leak was found. **(C)** Left ONSD just before dynamic myelography. ONSD was already moderately dilated by SIH to 6.4 mm (normal £ 5.8 mm). Transcranial Doppler ultrasonography findings were normal on both sides (not shown). **(D)** Left ONSD during dynamic myelography. ICP increased from 2 to 80 mmHg for a few seconds, then returned to a normal value within 5 min. ONSD dilation was further increased to 7.7 mm by intrathecal injection. The ONSD remained high, with maximal values in the following days. Only the left side is shown, but the same pattern was observed on the right side. **(E)** Phlebography–CT scan (D36). An excessively closed angle between the vein of Galen and the right sinus: 12° (angle marked in red). **(F)** Digital subtraction angiography–phlebography (D55). The excessively closed angle between the vein of Galen and the right sinus was confirmed. Nevertheless, venous flow was normal, and no therapeutic procedure could be performed. (CSF, cerebrospinal fluid; ONDS, optic nerve sheath diameter).

Supplementary Figure 2 Proposed algorithm for the investigation and therapeutic management of SDH with SIH: The therapeutic and etiological strategy is increasingly invasive: - initial evaluation by skull and spine MRI sequences- non-targeted lumbar epidural patches, possibly with saline, which may be repeated once or twice- in case of failure, dynamic myelography to search for a persistent extensive tear, with surgical repair if present.No indication for surgical removal of a subacute SDH except in cases of coma with uncus herniation.This algorithm follows logically from our observations (previous and current clinical notes) but still needs to be the subject of larger prospective studies.(SDH, subacute subdural hematoma; SIH, spontaneous intracranial hypotension)

Supplementary TABLE 1 Main laboratory data.
