# Supplementary figures and images for: Case report: Simultaneous measurement of intracranial pressure and lumbar intrathecal pressure during epidural patch therapy for treating spontaneous intracranial hypotension syndrome. Spontaneous intracranial hypotension or spontaneous intraspinal hypovolume?
Source: Front Neurol. 2024 Mar 21;15:1308462. doi: 10.3389/fneur.2024.1308462 (PMC10991849; doi:10.3389/fneur.2024.1308462)

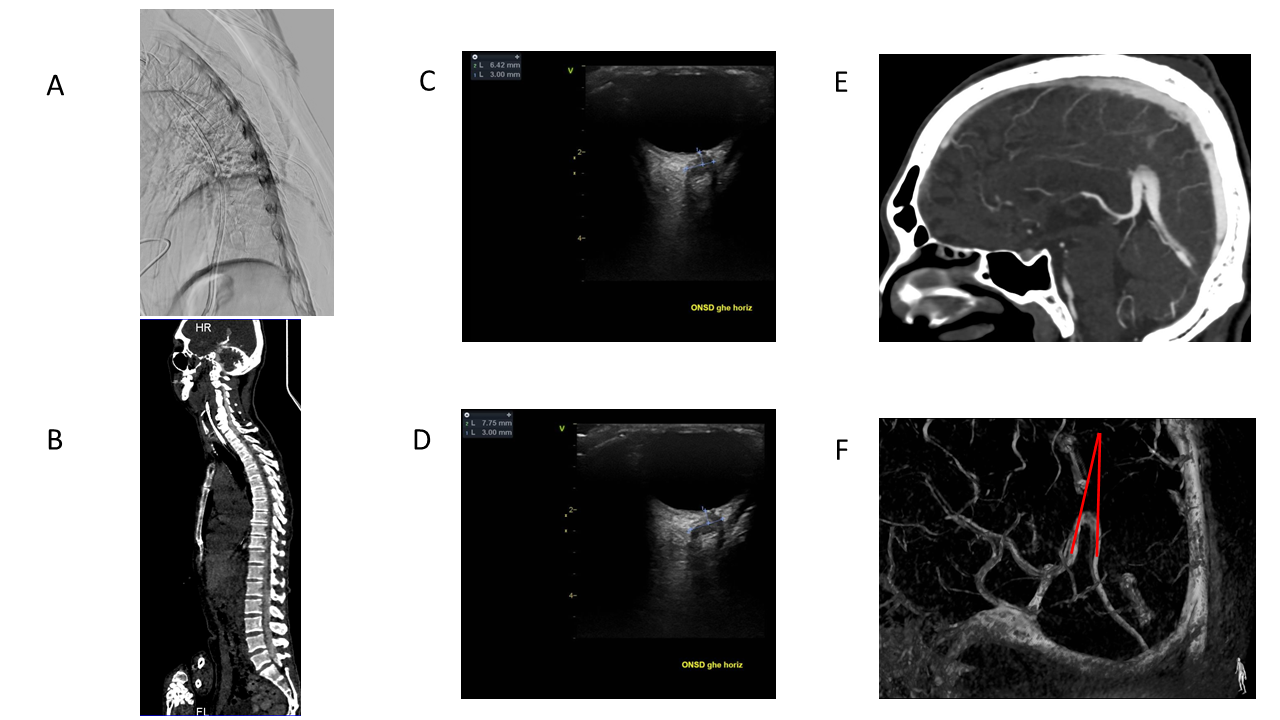

Supplement: Supplementary Figure 1 — Subsequent imaging. (A) First dynamic myelography (D25). No extra-thecal CSF leak found. (B) CT-scan myelography (D25). The patient was transferred for CT immediately after the dynamic myelography was performed, without further contrast injection into the intrathecal space. No extra-thecal CSF leak was found. (C) Left ONSD just before dynamic myelography. ONSD was already moderately dilated by SIH to 6.4 mm (normal £ 5.8 mm). Transcranial Doppler ultrasonography findings were normal on both sides (not shown). (D) Left ONSD during dynamic myelography. ICP increased from 2 to 80 mmHg for a few seconds, then returned to a normal value within 5 min. ONSD dilation was further increased to 7.7 mm by intrathecal injection. The ONSD remained high, with maximal values in the following days. Only the left side is shown, but the same pattern was observed on the right side. (E) Phlebography–CT scan (D36). An excessively closed angle between the vein of Galen and the right sinus: 12° (angle marked in red). (F) Digital subtraction angiography–phlebography (D55). The excessively closed angle between the vein of Galen and the right sinus was confirmed. Nevertheless, venous flow was normal, and no therapeutic procedure could be performed. (CSF, cerebrospinal fluid; ONDS, optic nerve sheath diameter). [file Image_1.tif]

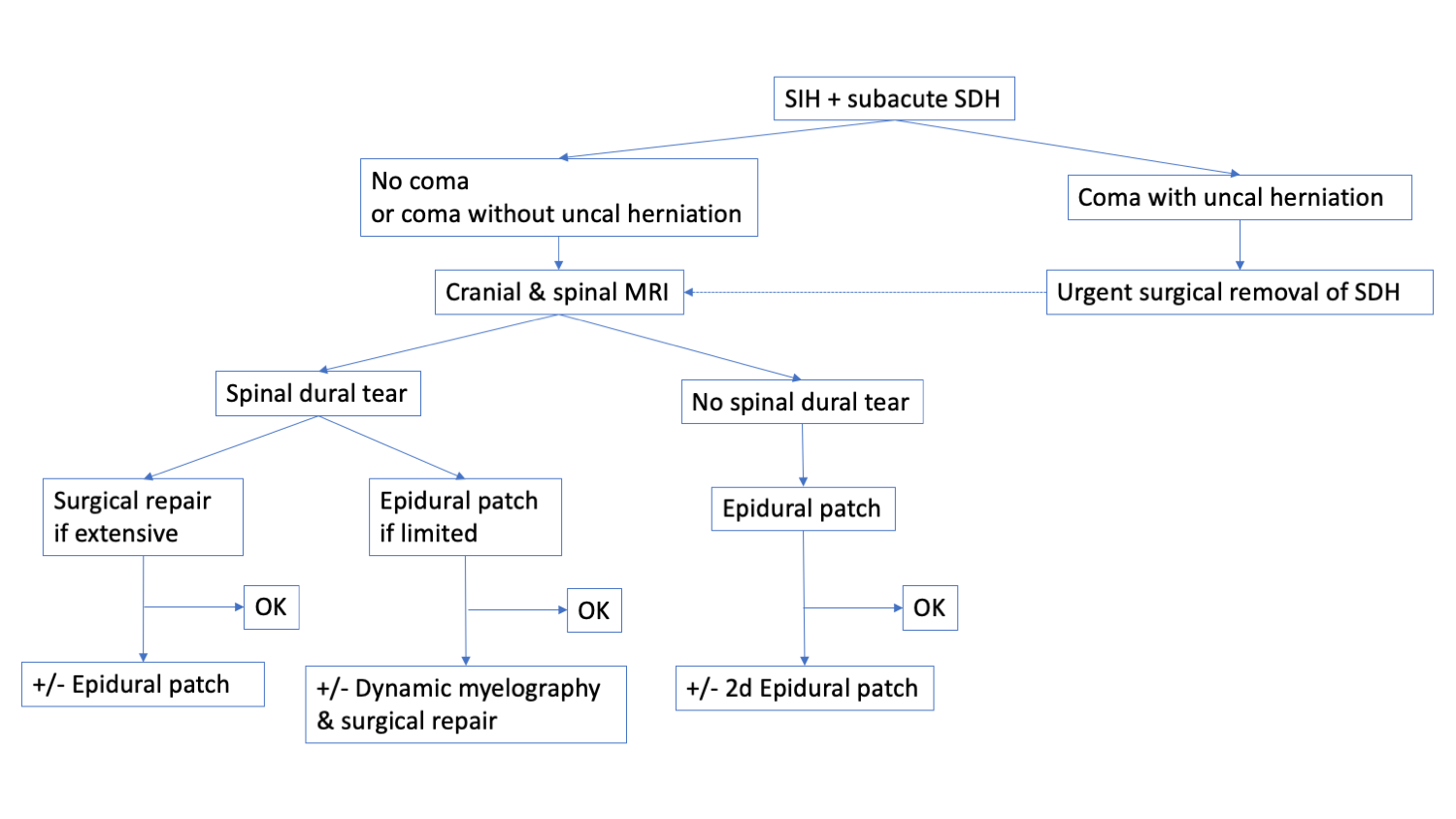

Supplement: Supplementary Figure 2 — Proposed algorithm for the investigation and therapeutic management of SDH with SIH: The therapeutic and etiological strategy is increasingly invasive: - initial evaluation by skull and spine MRI sequences- non-targeted lumbar epidural patches, possibly with saline, which may be repeated once or twice- in case of failure, dynamic myelography to search for a persistent extensive tear, with surgical repair if present.No indication for surgical removal of a subacute SDH except in cases of coma with uncus herniation.This algorithm follows logically from our observations (previous and current clinical notes) but still needs to be the subject of larger prospective studies.(SDH, subacute subdural hematoma; SIH, spontaneous intracranial hypotension) [file Image_2.tiff]
