# Supplementary material for: Case report: Simultaneous measurement of intracranial pressure and lumbar intrathecal pressure during epidural patch therapy for treating spontaneous intracranial hypotension syndrome. Spontaneous intracranial hypotension or spontaneous intraspinal hypovolume?
Source: Front Neurol. 2024 Mar 21;15:1308462. doi: 10.3389/fneur.2024.1308462 (PMC10991849; doi:10.3389/fneur.2024.1308462)
Supplement: Supplementary TABLE 1 — Main laboratory data. [file Table_1.DOCX]

**Supplementary material 1: Key laboratory data**

**Blood laboratory exams**

| **Biological parameter** | **D-4*** | **D1** | **D4** | **D12** | **D35** | **D40** |
| --- | --- | --- | --- | --- | --- | --- |
| Haemoglobin | 11.8 g/dL | 11.7 g/Dl |  |  |  |  |
| White blood count | 3.78 G/L | 6.16 G/L |  |  |  |  |
| Neutrophil polynuclear cells |  | 4.27 G/L |  |  |  |  |
| Eosinophil polynuclear cells |  | 0.5 G/L |  |  |  |  |
| Basophil polynuclear cells |  | 0.2 G/L |  |  |  |  |
| Lymphocytes |  | 0.98 G/L |  |  |  |  |
| Monocytes |  | 0.87 G/L |  |  |  |  |
| Platelet count | 207 G/L | 211 G/L |  |  |  |  |
| Prothrombin rate | 99 % | 94 % |  |  |  |  |
| Activated partial thromboplastin time | Ratio 1.05 | Ratio 1.00 |  |  |  |  |
| Sodium | 138 mmol/L | 135 mmol/L |  |  |  |  |
| Potassium | 3.8 mmol/L | 4.0 mmol/L |  |  |  |  |
| Chlorine |  |  |  |  |  |  |
| Urea | 5.1 mmol/L | 4.5 mmol/L |  |  |  |  |
| Creatinin |  | 76 mmol/L |  |  |  |  |
| Protein | 51 g/L | 79 g/L |  |  |  |  |
| Albumin | 37 g/L |  |  |  |  |  |
| Blood glucose |  | 5.0 mmol/L |  |  |  |  |
| Aspartate-Amino-Transférase | 40 UI/L | 47 UI/L |  |  |  |  |
| Alanine-Amino Transférase | 30 UI/L | 34 UI/L |  |  |  |  |
| gamma-glutamyltranspeptidase | 20 Ui/L | 21 UI/L |  |  |  |  |
| Alkaline phosphatase |  | 77 UI/L |  |  |  |  |
| B1 vitamin |  |  |  | 242nmol/l |  |  |
| HIV Western Blot | Positive |  |  |  |  |  |
| Hepatitis B virus serology | Negative |  |  |  |  |  |
| Hepatitis C virus serology | Negative |  |  |  |  |  |
| Total anti-syphilis antibodies |  | Negative |  |  |  |  |
| SARS-CoV-2 RT-PCR |  | Negative |  |  | Negative | Negative |
| Toxoplasma serology |  |  | Negative |  |  |  |
| CMV serology |  |  | IgG+  IgM- |  | IgG+  IgM- | IgG+  IgM- |
| HIV serology (1+2) |  |  | Positive |  |  | Positive |
| HIV-1 viral load |  |  | 5.4 log |  |  | 2.28 log |
| HIV antigen p24 |  |  | Negative |  |  |  |
| HIV Resistance genotype (RT and protease) |  |  | No mutation |  |  |  |
| CD 4 Lymphocyte count |  |  | 4/mm^3^ |  |  | 18/mm^3^ |
| CD4 / CD 8 ratio |  |  | 0.03 |  |  | 0.05 |
| Soluble Cryptococcus neoformans Antigen |  |  |  | Negative |  |  |
| Human herpesvirus type 8 PCR |  |  |  | Negative |  |  |
| Anti-neuronal blood antibodies** |  |  |  |  |  | Negative |
| Blood ammonia |  |  |  |  |  | 25 µmol/L |

* D-4: day of the arrival to the first hospital (day 0 = day of SDH surgical evacuation)

** Anti-neuronal blood antibodies: anti-Hu, anti-Yo, anti-Ri, anti-CV2, anti-amphiphysin, ant-Ma2, anti-SOX1, anti-GAD65, anti-Tr, anti-Zic4, anti-Titine

A culture of mycobacteria in the tracheal secretions was also performed on D12: negative

**CSF laboratory exams**

| **Biological parameter** | **Day 20** | **Day 36** | **D48** |
| --- | --- | --- | --- |
| White blood count | 39 /mm^3^ | 10 /mm^3^ |  |
|  | No lymphoma cells | No lymphoma cells |  |
| Neutrophil polynuclear cells |  |  |  |
| Lymphocytes | 99 % |  |  |
| Red blood count | 1098 /mm^3^ | 135 /mm^3^ |  |
| Protein | 1.57 g/L | 0.58 g/L |  |
| Glucose | 2.15 mmol/L | 2.34 mmol/L |  |
| Gram coloration | sterile | sterile |  |
| Bacterial culture | sterile | sterile |  |
| Multiplex PCR * | Negative | Negative |  |
| Herpes Simplex Virus 1&2 PCR |  |  | Negative |
| Cytomegalovirus PCR |  |  | Negative |
| Epstein-Barr Virus PCR |  |  | Negative |
| Enterovirus PCR |  |  | Negative |
| Human Herpesvirus 6 PCR |  |  | Negative |
| Human Herpesvirus 8 PCR |  |  | Negative |
| Polyomavirus JC PCR |  |  | Negative |

* Multiplex PCR:

- Virus: Cytomegalovirus (CMV), Enterovirus, Herpes Simplex Virus 1 (HSV1), Herpes Simplex Virus 2 (HSV2), Human Herpesvirus 6 (HHV-6), Human Parechovirus, Varicelle Zona Virus (VZV)

- Bacteria: Escherichia coli K1, Haemophilus influenza, Listeria monocytogenes, Neisseria meningitidis, Streptococcus agalactiae, Streptococcus pneumonia

- Levure: neoformans / gatti
